# Beyond Traditional and Nontraditional Roles: Reframing Health Information Practice Through the Health Information Continuum

**DOI:** 10.63116/AH.000000001

**Published:** 2026-06-10

**Authors:** Leah Grebner, Angela Campbell

**Affiliations:** 1 Department of Health Careers, Illinois Central College; 2 Department of Health Information Technology, San Juan College

**Keywords:** Health Information Continuum, health information workforce, professional identity

## Abstract

**Background:**

The health information (HI) profession has expanded beyond hospital-centered roles, yet it continues to rely on the labels “traditional” and “nontraditional,” which no longer reflect workforce realities.

**Methods:**

In this study, the authors used an evidence-informed, mixed-methods synthesis incorporating federal workforce data, professional literature, and original survey data from HI practitioners and educators.

**Results:**

The findings demonstrate that HI roles are increasingly cross-sector, technology-enabled, and functionally interconnected, with substantial workforce activity occurring outside hospital settings and beyond federally classified roles.

**Conclusions:**

In this article, the authors introduce the Health Information Continuum as a conceptual framework that reframes HI work around shared competencies and the health data life cycle, offering a more accurate and inclusive model for workforce description, education, and professional identity.

## Introduction

The health information (HI) profession has expanded far beyond the hospital-centered environments that once defined its scope. Despite this evolution, the profession continues to rely on the labels “traditional” and “nontraditional” to describe roles and career pathways. Although these terms originated as descriptive shorthand, they no longer reflect how HI work is organized, practiced, or experienced across the current health care ecosystem. Their continued use contributes to misalignment between professional identity, workforce realities, and educational preparation.

In this article, the authors examine the historical origins and unintended consequences of setting-based language in HI practice and synthesize workforce evidence demonstrating its growing inadequacy. Drawing on federal workforce data, professional literature, and original survey findings from practitioners and educators, this research introduces the Health Information Continuum as an alternative framework for understanding HI work. Rather than organizing roles by setting, the continuum frames practice around shared competencies and the life cycle of health data. Part I provides historical context, followed by workforce evidence, methodological approach, survey findings, and presentation of the continuum model mentioned in Parts II-VI.

## Part I: Background and Problem Statement

### Historical Context: How the “Traditional” and “Nontraditional” Labels Developed

The distinction between “traditional” and “nontraditional” roles in HI emerged during a period when the profession was almost entirely embedded within hospitals and clinical settings. Early HI work centered on medical records departments responsible for documentation, coding, release of information, and chart management, all within paper-based and facility-specific workflows. As formal credentialing and accredited education programs developed, acute care environments became the default reference point for defining HI practice and professional identity.^1^

As the health care system evolved, new opportunities for HI professionals emerged beyond hospital settings, including roles in physician practices, insurance organizations, government programs, registries, and early HI technology environments. Because these positions fell outside the original hospital-based model, they were described as “nontraditional,” a term that initially served as a convenient shorthand to distinguish newer settings from the established norm.^1^ Over time, however, this binary language became embedded in professional discourse, educational materials, and workforce conversations, solidifying into a persistent framework for categorizing HI roles even as the scope of the profession continued to expand across increasingly diverse sectors.^2^^,^^3^

### The Unintended Hierarchy Created by Setting-Based Language

This framing has shaped expectations about what HI work is supposed to look like. Over time, the language shifted from describing where early work occurred to signaling which roles were viewed as core vs peripheral. This shift has influenced how educational programs describe career options, how students conceptualize professional pathways, and how practitioners perceive the value and visibility of their own work.^4^

### Emerging Complexity in HI Practice

The expansion of digital health, interoperability initiatives, analytics, and data-driven care models has increasingly blurred the boundaries between traditional health information management (HIM) and informatics, with HI professionals now contributing to roles that require technical, analytical, and governance expertise in addition to foundational HIM competencies—many of which did not exist when the traditional-nontraditional distinction first emerged.^2^^,^^5^ Across provider, payer, vendor, public health, research, education, and government environments, these professionals support data quality, privacy, interoperability, identity management, analytics, and regulatory compliance—responsibilities that operate across organizational boundaries and rely on coordinated data flows rather than isolated record systems. As a result, HI work is best understood as an interconnected ecosystem rather than a collection of discrete, setting-based roles.^3^^,^^6^

### Problem Statement

Addressing this disconnect requires a clearer understanding of how the profession has evolved, why existing labels persist, and how alternative frameworks might better represent the full scope of HI work. The following section examines workforce evidence and structural limitations that demonstrate why the traditional-nontraditional framing is no longer sufficient.

## Part II: The Case for Change

The continued use of “traditional” and “nontraditional” language no longer aligns with the realities of modern HI practice. Although historically grounded, this binary framework oversimplifies the profession, obscures the breadth of roles that now exist, and reinforces outdated assumptions about where HI work occurs and how it should be valued.^2^^,^^3^

As HI responsibilities expand across sectors, there is a growing disconnect between professional language and workforce reality. This misalignment affects how the profession is understood by students, educators, employers, policymakers, and even HI professionals themselves.^4^^,^^6^

### Why the Traditional and Nontraditional Model Is Outdated

Although the labels “traditional” and “nontraditional” were not intended to create hierarchy, they emerged during a period when hospital-based work defined HI practice and positioned hospital and clinic roles as the profession’s central reference point. As HI responsibilities expanded into distributed, technology-enabled, and cross-sector environments, including governance, interoperability, analytics, compliance, and population health, the binary framework failed to evolve and became increasingly strained. Yet roles in these environments are still often framed as deviations rather than recognized as integral components of the workforce. This misalignment reflects the limitations of setting-based labels, which can no longer capture the breadth and interdisciplinary nature of modern HI practice, reinforcing the need for updated frameworks that reflect how HI work functions across interconnected systems rather than isolated locations.^2^^,^^3^^,^^5^^,^^7^

### Evidence That the Workforce Now Works Beyond Hospital Walls

Federal workforce data provide an important, though incomplete, lens for examining employment patterns within HI roles. Bureau of Labor Statistics (BLS) data for Standard Occupational Classification (SOC) 29-2072 Medical Records Specialists indicate that approximately 28% of individuals classified under this code are employed in hospital settings, whereas the remaining majority work in physician offices, management services, administrative support services, and other nonhospital environments. BLS data for SOC 29-9021 Health Information Technologists and Medical Registrars similarly indicate that more than half of individuals classified under this code are employed outside hospital systems.^8^^,^^9^

When SOC 29-2072 and SOC 29-9021 are examined together, the combined weighted industry distribution indicates that hospital employment accounts for approximately 31% of the federally classified workforce, reinforcing that hospital-based employment is no longer the dominant setting even within the limited portion of the workforce captured by federal occupational classifications.^8^^,^^9^

Table 1 summarizes where federally classified HI roles are employed, using a combined weighted distribution across 2 SOC codes. It is presented to illustrate relative concentration by industry, not to define the full HI workforce.^8^^,^^9^

**Table 1.** Distribution of Health Information Professionals by Industry.

Industry Sector	Percent of Workforce (%)	Notes
Hospitals (state, local, private)	31	Acute care, specialty, integrated delivery systems
Offices of physicians	17	Ambulatory care, group practices, outpatient clinics
Management companies and enterprises	8	Corporate services, administrative management, shared services
Administrative and support services	7	Billing firms, outsourced medical records services, compliance vendors
Professional, scientific, and technical services	7	Consulting firms, legal services, analytics firms, auditing services
All other industries	30	Insurance carriers, payers, public health, education, research, registries, government, technology vendors, long-term care, emerging sectors
Total	100	Combined weighted distribution

Data synthesized from Bureau of Labor Statistics.^8^^,^^9^ Combined weighted distribution for SOC 29-9021 and 29-2072.

#### Interpretation

Table 1 demonstrates that even within federally classified HI roles, most employment occurs outside hospitals. The “all other industries” category represents a residual grouping of industries not otherwise specified within this distribution and should not be interpreted as a precise measure of employment in specific sectors such as payers, public health, education, research, registries, government, or technology vendors. The table demonstrates that hospital-centric assumptions no longer hold even within conservative, classification-bound datasets.^8^^,^^9^

Although Table 1 helps clarify employment patterns within federally classified roles, it also highlights a deeper problem. Many HI roles central to the current data ecosystem fall entirely outside these classifications.^2^^,^^3^

### Structural Limitations in Workforce Classification Systems

Workforce distribution patterns reveal a deeper structural issue in that existing occupational classification systems capture only a narrow subset of HI roles while excluding many positions central to the current health data ecosystem. Roles across payer, vendor, public health, research, education, and government environments are often not represented in current SOC codes, despite that large segments of HI work now occur in functions such as risk adjustment, claims analytics, fraud and abuse detection, interoperability, identity management, data governance, and digital health infrastructure. Although these roles rely on core HI competencies, they remain structurally invisible within commonly cited federal datasets, resulting in workforce descriptions that can misrepresent both the size and diversity of the profession.^2^^,^^3^^,^^6^

Table 2 illustrates the gap between what federal occupational codes capture and the broader set of roles that rely on HI competencies across the health care ecosystem.^2^^,^^3^

**Table 2.** Scope of BLS Occupational Codes Compared With the Broader HI Workforce.

Included in BLS codes (29-2072 and 29-9021)	Not included but integral to the HI workforce
Medical records specialists	Payers and risk-bearing entities (Medicaid, Medicare advantage, commercial plans, TPAs, PBMs)
Health information technologists and registrars	Vendors and technology companies (EHR developers, HIEs, analytics firms, AI developers, digital health platforms)
Coding and documentation roles	Consulting and revenue cycle firms (auditing, compliance, risk adjustment, project management, data governance)
Limited administrative and support services	Public health and registries (CDC, state/local health departments, cancer and trauma registries, vital records)
	Education and workforce development (faculty, program directors, instructional designers, certification developers)
	Research and life sciences (clinical trials, IRBs, data curation, life-science informatics)
	Policy, legal, and professional associations (CMS, ONC, VA/DoD, OIG, AHIMA, IFHIMA)
	Animal and emerging health fields (veterinary hospitals, zoos, biotech, device companies)
	Other emerging roles (global health, defense systems, innovation startups, health communication, patient-education media)

Abbreviations: BLS, US Bureau of Labor Statistics; HI, health information; AI, artificial intelligence; CDC, Centers for Disease Control and Prevention; TPA, Third Party Administrator; PBM, Pharmacy Benefit Manager; EHR, Electronic Health Record; HIE, Health Information Exchange; IRB, Institutional Review Board.

#### Interpretation

Table 2 highlights the limits of federal occupational coding systems, which recognize only a narrow subset of job categories commonly associated with HI work. Large segments of the workforce, especially those in payer, vendor, public health, research, and education environments, are excluded from official classifications even when these roles rely heavily on HI competencies. This structural invisibility contributes to persistent mischaracterization of the profession and reinforces the inadequacy of a binary setting–based model.^2^^,^^3^

### Impact of Language on Perception, Recruitment, and Career Identity

Beyond workforce distribution and classification systems, the language used to describe HI roles has tangible consequences for how the profession is perceived and valued. Terminology such as “nontraditional” can imply marginal status and reinforce outdated assumptions about where HI work belongs, contributing to misunderstandings of roles in payer organizations, vendor firms, public health agencies, analytics companies, and government programs—despite their central importance to health data operations. This disconnect shapes how students select educational pathways, how educators frame the profession, and how employers assess the value of HI competencies. Although the AHIMA Career Map illustrates diverse pathways across governance, informatics, analytics, privacy, policy, education, and research, many of these roles remain excluded from what has historically been labeled “traditional,” underscoring the need to update workforce language to strengthen professional identity and make the full range of career opportunities visible.^2^^,^^4^^,^^6^^,^^10^

### Comparison to Other Industries That Have Evolved Beyond Setting-Based Definitions

Other professions have faced similar identity shifts as their work expanded beyond a single setting. Information technology moved from on-site support to distributed roles across industries. Public health transformed from field epidemiology to data-driven surveillance, informatics, policy analysis, and global health. In each case, professional vocabulary evolved to reflect the scope and function of work rather than physical location.^3^

A similar transition is underway in HI. The profession is increasingly defined not by where professionals work but by how they steward, govern, exchange, analyze, and apply health data to improve outcomes. Together, workforce distribution patterns, classification limitations, and professional identity impacts reinforce the need for a new model that aligns with the actual breadth and function of the HI profession.^2^^,^^3^^,^^6^

The following section describes the approach used to develop a model that reflects how HI work actually functions across the health care ecosystem.

## Part III: Methods for Developing the Continuum Model

### Ethical Considerations

This study was reviewed and determined not to meet the definition of human subjects research and, therefore, did not require institutional review board approval. Survey data were collected anonymously and reported in aggregate.

A structured survey instrument was distributed to Illinois Health Information Management Association (ILHIMA) members (*n* = 117 respondents) and Illinois HI/HIM program directors (*n* = 11 respondents). Surveys included items on work setting, job function, education, credentials, and perceptions of career pathways. Response data were analyzed descriptively to identify workforce patterns, career mobility, and alignment with educational preparation.

The Health Information Continuum was developed using a mixed-methods, evidence-informed synthesis approach that integrates workforce data, educational perspectives, and real-world practice patterns into a conceptual model. Because no single dataset captures the full scope of HI roles across sectors, the methodology emphasizes triangulation across multiple quantitative and qualitative sources rather than reliance on a single empirical measure.^2^^,^^11^^,^^12^

Publicly available datasets from the US BLS and federal health workforce analyses were reviewed to establish baseline employment distributions for HIM- and HI-adjacent occupations. These sources provided anchoring insight into provider-based roles while highlighting limitations in capturing emerging, hybrid, and non-provider-based positions.^8^^,^^9^^,^^11^

Primary data collected through surveys of Illinois HI professionals and HI/HIM program directors were used to contextualize workforce distribution, career mobility, role fluidity, and educational exposure. These findings informed the assessment of alignment between the conceptual model and lived professional experience.

The final Health Information Continuum consists of 10 major sectors that represent the full range of environments in which HI professionals contribute, illustrating how HI is created, managed, protected, analyzed, exchanged, and used across clinical, administrative, public health, educational, research, and emerging contexts. Developed to capture the breadth of employment settings not fully reflected in any single dataset, the model synthesizes multiple sources, including federal labor data, national workforce surveys, employer ecosystem analyses, and job-market evidence. The continuum includes 10 sectors: provider and care delivery, payers and risk-bearing entities, vendors and technology organizations, consulting and revenue cycle firms, public health and registries, education and workforce development, research and life sciences, policy and professional associations, animal and emerging health fields, and other emerging roles.^2^^,^^3^

Proportional estimates for the model were informed by BLS industry distributions for HI-related occupations, workforce totals across the insurance and payer ecosystem, Healthcare Information and Management Systems Society employer distributions for informatics and health IT professionals, federal Health Information Technology (HIT) workforce evaluations from Health Resources and Services Association’s National Center for Health Workforce Analysis, and Office of the National Coordinator’s national HIT workforce analyses.^8^^,^^9^^,^^11–15^

### Limitations

The Health Information Continuum reflects the best available evidence but is constrained by persistent gaps in workforce data, as existing sources capture only portions of the profession. BLS occupational codes represent a narrow subset of HI roles and exclude many positions in informatics, payer organizations, technology vendors, public health agencies, education, research, and emerging digital health environments. Some national datasets emphasize informatics professionals rather than the full HI workforce, insurance-sector data include roles that overlap with HI competencies without being exclusively classified as HI positions, and job postings indicate employer demand but do not measure workforce size.^8^^,^^9^^,^^11–13^^,^^15^ For these reasons, the continuum should be understood as an evidence-informed conceptual model rather than a definitive census, synthesizing federal, industry, and market-based data to illustrate the scope and distribution of HI roles. It is not intended to serve as a credentialing schema, job taxonomy, or replacement for existing role-based tools, but as a complementary framework for understanding how HI work is organized and interconnected across the health care ecosystem.

## Part IV: Survey Findings: Practitioner and Educator Perspectives

This section presents practitioner and educator perspectives to illustrate how HI professionals experience role diversity, career mobility, and setting boundaries in practice and education. The findings are descriptive and intended to contextualize workforce patterns and perceptions rather than to generalize nationally. Interpretation of these findings and their implications for professional identity and workforce framing are addressed in the following section.

### Survey of ILHIMA Membership

A general membership survey was distributed to ILHIMA members, yielding a total of 117 responses. Substantively, the data indicate a heavily tenured workforce, with more than half of respondents reporting over 20 years in the field and an additional quarter reporting 11-20 years of experience.

Beyond retirement risk, longevity in the field may also be associated with skill stagnation if ongoing professional development has not kept pace with rapid changes in technology, data governance, analytics, interoperability, and nontraditional HI roles. Many long-tenured professionals were educated before emerging settings such as vendor organizations, analytics, AI-enabled tools, payer environments, and remote work models became prevalent, potentially limiting awareness of or readiness for these opportunities. These concerns align with broader workforce findings on role expansion and the increasing influence of emerging technologies.^2^^,^^5^^,^^10^

Additional longevity-related concerns include burnout, resistance to role evolution or organizational change, limited succession planning, and challenges in mentoring newer professionals if career pathways are not clearly articulated. Collectively, these factors highlight the need for intentional upskilling, structured knowledge transfer, and targeted strategies to support both workforce renewal and the long-term sustainability of the HI profession.^2^

Several significant observations emerge from the education-level data that have important implications for the HI workforce and for how career pathways are framed. The majority of respondents hold at least a baccalaureate degree, with more than one-third reporting a bachelor’s degree and nearly one-quarter holding a master’s or doctoral degree, reflecting a highly educated professional population. At the same time, nearly one-third of respondents reported an associate degree as their highest level of education, underscoring the continued importance of associate-level entry points into the profession. The relatively small proportion of certificate-only respondents and the absence of high school-only pathways suggest that formal postsecondary education remains a foundational expectation in HI roles. Taken together, these findings highlight a vertically diverse workforce in which multiple education levels coexist across roles and settings, reinforcing the need to move away from rigid role classifications and toward a continuum-based model that aligns competencies, career mobility, and lifelong learning opportunities rather than defining roles by degree level or labeling them as “traditional” or “nontraditional.”^2^

The credentialing data further illustrate the breadth and diversity of the current HI workforce and reinforce the limitations of labeling roles as “traditional” or “nontraditional.” Nearly half of respondents hold the Registered Health Information Administrator credential, and more than one-third hold the Registered Health Information Technician credential, demonstrating a strong foundation in core HIM competencies. Coding credentials remain prevalent, with Certified Coding Specialist and Certified Coding Association represented, but they do not dominate the credential landscape, indicating that HI practice extends well beyond coding-focused roles. Notably, a meaningful proportion of respondents hold credentials from other professional organizations or specialized certifications in areas such as privacy, compliance, revenue cycle, and practice management, reflecting cross-disciplinary career pathways and role evolution. Collectively, these findings support a continuum-based view of professional practice in which competencies span multiple settings and functions rather than fitting neatly into “traditional” vs “nontraditional” categories.^2^

The distribution of primary job functions reflects both the depth and complexity of contemporary HI roles when all responses are fully integrated. Coding remains the most frequently reported function, followed by leadership and administrative roles, indicating significant career progression and organizational responsibility. Education and training, revenue cycle and reimbursement, privacy, security, and compliance, IT systems or Electronic Health Record management, data governance and information management, and consulting or project-based work are also represented, demonstrating the breadth of operational, technical, and strategic functions performed by HI professionals. These patterns align with national findings describing expanding role expectations and technology-driven changes in work.^2^^,^^5^^,^^10^

The primary work setting data demonstrate a strong concentration of HI professionals within hospital and health system environments. Physician offices and educational institutions account for smaller shares, with representation in behavioral health, insurance or payer organizations, consulting and professional services, vendors, revenue cycle management firms, and government settings. Despite this breadth, many settings often described as “nontraditional,” including public health agencies, health IT vendors, telehealth, research, registries, and alternative care environments, are minimally represented. Collectively, these findings highlight the continued dominance of hospital-based practice while also illustrating the diversity of provider-adjacent and organizational settings in which HI professionals work, reinforcing the need to frame careers along a continuum rather than relying on narrow or binary classifications of work settings.^2^

The finding that more than half of respondents have worked in more than 1 type of setting is significant because it provides strong evidence of career mobility and role fluidity within the HI profession. This experience across multiple settings demonstrates that HI competencies are highly transferable and not confined to a single environment or narrowly defined role. It reinforces the concept of the Health Information Continuum by showing that professionals routinely move across care delivery, administrative, educational, payer, and other organizational contexts while applying a common foundation of skills in data governance, documentation, compliance, and information use. This mobility also challenges the usefulness of “traditional” vs “nontraditional” role distinctions, as many careers naturally evolve across settings over time.^4^^,^^10^

### Survey of Illinois Program Directors

Program directors from certificate, associate, baccalaureate, and graduate HI/HIM programs across the state of Illinois were surveyed, resulting in 11 total responses.

The educator survey responses indicate that hospital or acute care and ambulatory or physician office settings are universally included in curricula, reflecting their continued prominence in HI education. Beyond these core environments, respondents reported incorporating a wide range of additional settings, including long-term care, payer and insurance organizations, vendors, consulting, public health, research, education, government organizations, and veterinary settings. Collectively, these findings suggest that although traditional care delivery settings remain foundational, educators are increasingly exposing students to the broader continuum of HI practice across diverse organizational contexts.

More than one-third of program directors reported that their programs only minimally or somewhat reflect the full range of environments in which HI professionals work, indicating a meaningful gap between workforce reality and educational exposure. Although many programs acknowledge diverse HI career settings, factors such as curriculum constraints, limited resources, and restricted access to experiential learning appear to hinder deeper integration of non-provider-based environments. Program directors most often reported using assignments or case studies to introduce emerging HI career pathways, with fewer programs incorporating dedicated courses or modules, longitudinal integration across multiple courses, guest speakers, or experiential learning opportunities. Taken together, these findings suggest that although efforts are being made to expose students to evolving roles, the depth and consistency of that exposure vary widely, reinforcing the need for expanded frameworks, instructional resources, and partnerships that support a more systematic, continuum-based approach to HI education.

## Part V: Implications and Interpretation

This section interprets the workforce evidence and survey findings presented in the preceding sections and examines their implications for professional identity, competencies, and the framing of HI work.

The Health Information Continuum can be defined as a unified model encompassing all roles, settings, and specializations that manage, protect, and use health data to improve outcomes.

This workforce longevity finding directly reinforces the thesis of moving away from labeling roles as “traditional” vs “nontraditional” and instead adopting a continuum-based view of HI careers. A highly tenured workforce may have implications for workforce sustainability, including potential impacts on capacity, knowledge transfer, and leadership continuity. The concentration of long-tenured professionals, many educated and socialized into a provider-centric model of HI practice, helps explain why certain roles continue to be perceived as “nontraditional,” even though they are now well established and critical to the health care ecosystem. These labels are less reflective of the roles themselves and more indicative of when and where professionals entered the field and how HI education historically framed career pathways.^2^^,^^10^

By reframing HI work along a continuum of data creation, management, protection, analysis, exchange, and use across multiple settings, the profession can better acknowledge that all roles are valid, evolving, and interconnected. This approach supports workforce sustainability by encouraging lifelong learning, facilitating reskilling for experienced professionals, and helping newer entrants view the full breadth of career opportunities as integral, not alternative, to the core of HI practice.^11^

The synthesis approach for the Health Information Continuum integrates multiple perspectives, roles, and functions across health care and health-related domains into a single, cohesive framework. Rather than segmenting work into isolated or hierarchical categories, the approach deliberately blends practice settings, competencies, and data uses to reflect how HI actually flows and creates value. The continuum is synthesized using a systems-based perspective that views HI as an interconnected life cycle rather than discrete tasks or job titles. This prevents artificial boundaries between clinical, administrative, and nonclinical roles.^3^^,^^10^

Competencies such as data integrity, privacy, interoperability, analytics, ethics, and governance are interwoven across disciplines rather than assigned to a single profession. This approach highlights transferable skills and shared accountability, reinforcing that no single role owns HI.^4^^,^^6^

The responses collectively reflect a strong desire for greater recognition and understanding of the complexity and value of HI work, while also reinforcing the central themes of the Health Information Continuum and the need to move beyond labeling roles as “traditional” or “nontraditional.” Respondents noted that the scope and impact of HI work are frequently misunderstood, with many assuming it is limited to discrete tasks such as data entry or error correction. In reality, HI professionals operate across the continuum, supporting data integrity, documentation quality, charge capture, compliance, and the effective use of increasingly automated systems, regardless of setting or job title. The comments also emphasize the specialized nature of this work and the necessity of continuous education to keep pace with evolving technologies, regulations, and workflows. Collectively, these insights portray HI professionals as an essential yet often invisible backbone of health care operations and underscore that their contributions are best understood through a continuum-based lens that recognizes interconnected responsibilities, evolving competencies, and the full breadth of professional impact across the health care ecosystem.^2^^,^^10^

The workforce development and education are directly connected to practice realities. Academic preparation, continuing education, and career mobility are embedded into the continuum to show how professionals move fluidly across roles and settings over time, supporting lifelong learning rather than linear career paths.^11^^,^^12^

The best approach is to describe the workforce as clusters within the Health Information Continuum, rather than silos of different settings. The clusters may be represented as providers and care delivery, payers revenue cycle risk management and utilization, public health, data and analytics, education training and workforce development, and consulting.^3^

Across the Health Information Continuum, within these clusters, it is important to recognize the fact that HI professionals rely on the same foundational principles for information governance of accountability, transparency, integrity, protection, compliance, availability, retention, and disposition.^16^

Finally, the continuum aligns all components around outcomes, including improved individual care, population health, equity, compliance, and system performance, consistent with federal strategic priorities for health system transformation.^17^ This outcome orientation ensures that the model is not merely descriptive, but strategic, demonstrating how diverse roles collectively contribute to measurable health improvement.

## Part VI: Introducing the Health Information Continuum

The Health Information Continuum is a unified framework designed to represent the full scope of HI work across settings, roles, and functions. Rather than organizing the profession by traditional care environments, the continuum describes HI practice as an interconnected system of data creation, management, exchange, analysis, and use that spans the entire health care ecosystem.^2^^,^^3^ The Health Information Continuum is intentionally nonlinear. It does not represent a sequential career ladder or progression of roles but rather a dynamic and interconnected system in which professionals may enter, move across, and contribute within multiple sectors and functions over time.

The model is intentionally role-agnostic, focusing on functions rather than titles, setting-inclusive, incorporating provider and non-provider environments, life cycle-based, aligned to the flow of health data, and outcome-oriented, emphasizing impact on care quality, population health, equity, compliance, and system performance.^3^

### Purpose of the Model

The Health Information Continuum provides a shared framework and common language for describing the full scope of HI work across the life cycle of health data, from creation and capture through use and outcomes. It connects long-standing HIM functions with emerging roles in informatics, analytics, data governance, digital health, population health, and consumer engagement within a single conceptual model. By acknowledging workforce diversity, career mobility, and interdisciplinary practice, the continuum supports more accurate workforce representation, clearer career pathways, and a more inclusive professional identity in which all HI roles are understood as integral to health care operations and outcomes.^2^^,^^10^

Having outlined the purpose of the Health Information Continuum, the discussion now turns to the structure of the model and the relationships among its core components.

### Why It Matters

The continuum underscores a lifelong responsibility for improving the quality, accessibility, and ethical use of health data. This emphasis aligns with AHIMA’s mission of “empowering people to impact health,” which reflects the profession’s commitment to ensuring that HI is complete, available, and protected across all environments where data are created or used.^18^

This perspective encourages professionals to adapt as health care evolves, pursue ongoing education, and contribute to emerging areas such as digital health, AI oversight, and population health.^10^

By seeing themselves along a continuum rather than within siloed roles, HI and broader HI professionals gain a clearer understanding of how their contributions support both individual patient care and global health outcomes. For example, accurate documentation and coding improve clinical decision-making at the point of care, whereas high-quality population-level data fuel public health surveillance, research, and international health initiatives.^19^^,^^20^

Recognizing these connections strengthens collaboration and reinforces the idea that every function, whether in a hospital, payer organization, technology company, educational institution, or public health agency, advances the shared goal of improving health through information.^3^

This model responds directly to the limitations identified earlier in this article by reframing HI work around purpose and function rather than employment setting. As roles have expanded beyond hospital and clinic walls, setting-based labels such as “traditional” and “nontraditional” no longer reflect how professionals contribute or how health data moves across organizations. Instead of anchoring the model to location, the Health Information Continuum synthesizes hospitals, physician practices, payers, vendors, public health agencies, educators, researchers, correctional facilities, and community-based organizations into a single framework based on their shared interaction with HI. By aligning these environments to the full health data life cycle, from creation and capture through governance, protection, analytics, exchange, and outcomes, the continuum illustrates how decisions made upstream directly influence downstream uses such as population health, reimbursement, research, and policy.^2^^,^^3^

**Figure 1. The Health Information Continuum.**
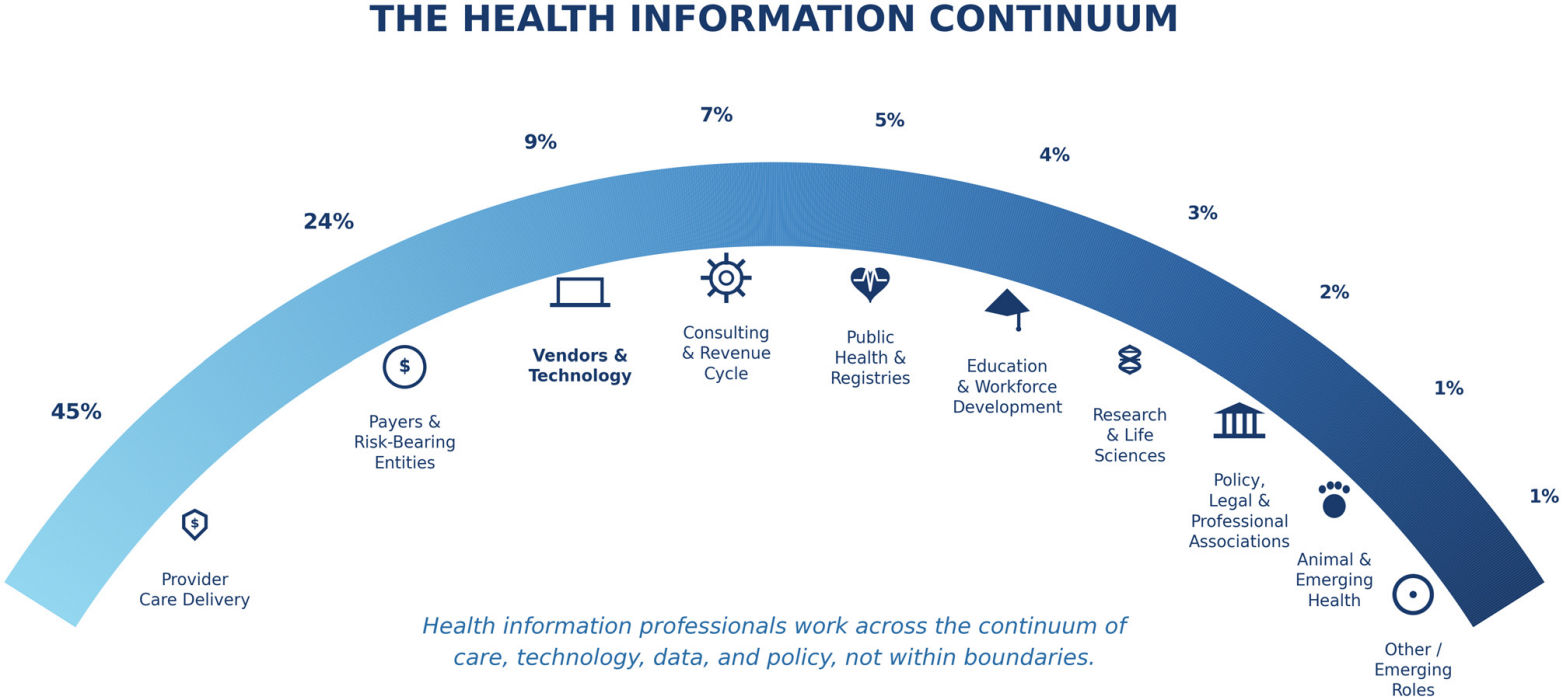


The Health Information Continuum illustrates the breadth of environments in which HI professionals work across care delivery, technology, data, policy, and emerging fields (Figure 1). Sector proportions are illustrative and represent synthesized multisource estimates used to convey scope rather than definitive workforce counts.^5^^,^^8^^,^^9^^,^^11–15^

### How the Continuum is Structured

The Health Information Continuum is best understood through 3 complementary lenses rather than as a single linear pathway.

First, sectors describe where HI work occurs. These sectors include provider and care delivery organizations, payers and risk-bearing entities, vendors and technology organizations, consulting and revenue cycle firms, public health and registries, education and workforce development, research and life sciences, policy and professional associations, animal and emerging health fields, and other evolving environments. Together, these sectors represent the settings across which health data are created, managed, and used.^3^

Second, core elements describe what happens to health data over time. Across all sectors, HI work follows a shared life cycle that begins with data creation and capture; continues through data management and protection, data exchange and integration, and data analysis and application; and culminates in data use and outcomes that support decision-making and improve health.^19^^,^^20^

The Health Information Continuum emphasizes that health data are created, managed, protected, analyzed, exchanged, and used across multiple settings, with each stage influencing the next. Rather than functioning as isolated steps, these activities are interdependent, meaning decisions made early in the data life cycle directly shape downstream outcomes related to care quality, reimbursement, population health, research, and policy. Viewing HI work through this life cycle lens highlights the shared responsibility of professionals across roles and environments for ensuring that data remain accurate, trustworthy, and usable throughout its journey.^19^^,^^20^

**Figure 2. The Health Information Continuum Core Elements.**
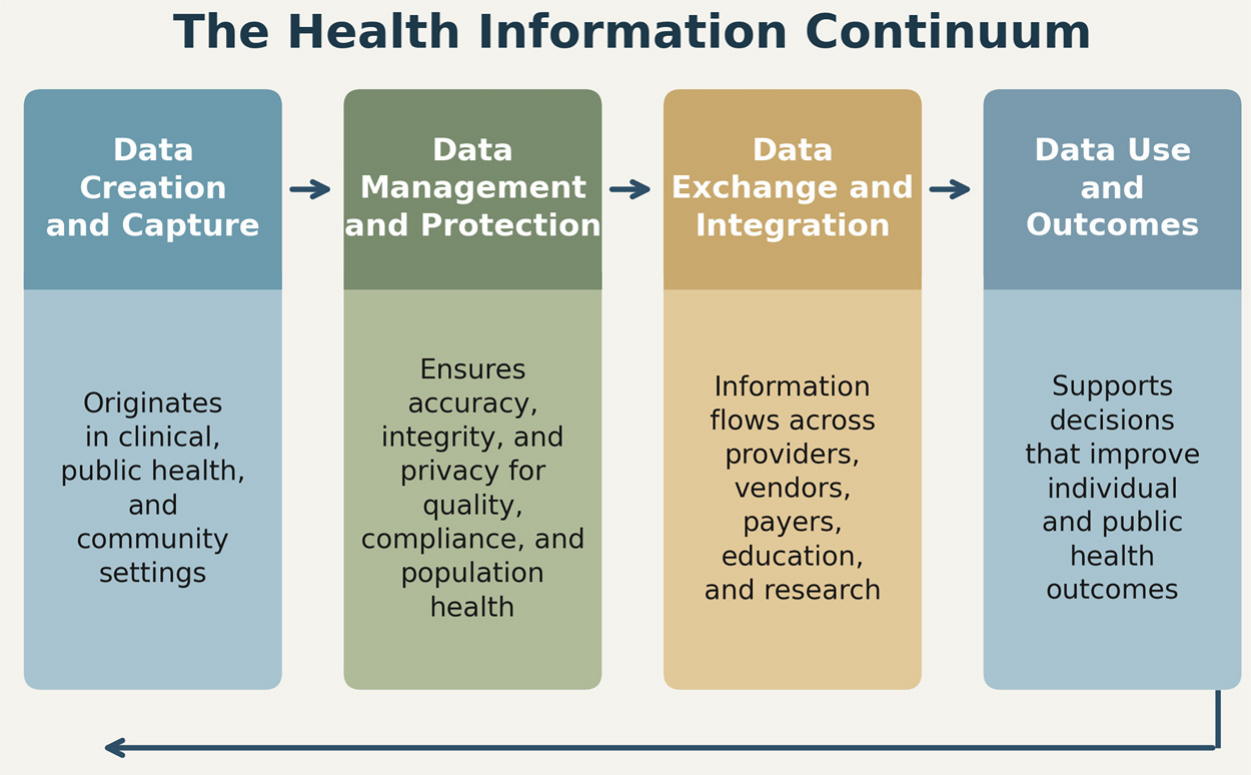


Core elements of the Health Information Continuum showing the life cycle of health data from creation through use and outcomes across all settings (Figure 2).^19^^,^^20^

Third, roles describe how professionals contribute within and across sectors. HI professionals may focus on governance, privacy, and compliance, documentation, coding, and revenue integrity, analytics, informatics, and technology, public and population health, or education, research, and workforce development, as reflected in the AHIMA Career Map.^7^ Many professionals work across multiple roles and settings over the course of their careers, reinforcing the need for a model that supports mobility rather than rigid classification.^4^

The continuum is intended to complement, rather than replace, existing role- and competency-based frameworks by providing a broader structural context in which such tools can be situated. The Health Information Continuum reframes HIM as a dynamic, interconnected ecosystem rather than a collection of isolated job functions. As emphasized in AHIMA’s Information Governance Principles for Healthcare, high-quality HI is foundational to safety, equity, and operational efficiency and depends on coordinated stewardship across roles and settings.^21^

The continuum highlights that roles such as coding, informatics, data governance, privacy, analytics, and public health data stewardship do not operate independently. Instead, they rely on shared competencies such as data accuracy, integrity, interoperability, and ethical stewardship. Viewing HI in this integrated way aligns with AHIMA’s Information Governance principles, which stress coordinated processes to support trustworthy, usable information throughout its life cycle.^21^

**Figure 3. The Health Information Continuum Role Clusters.**
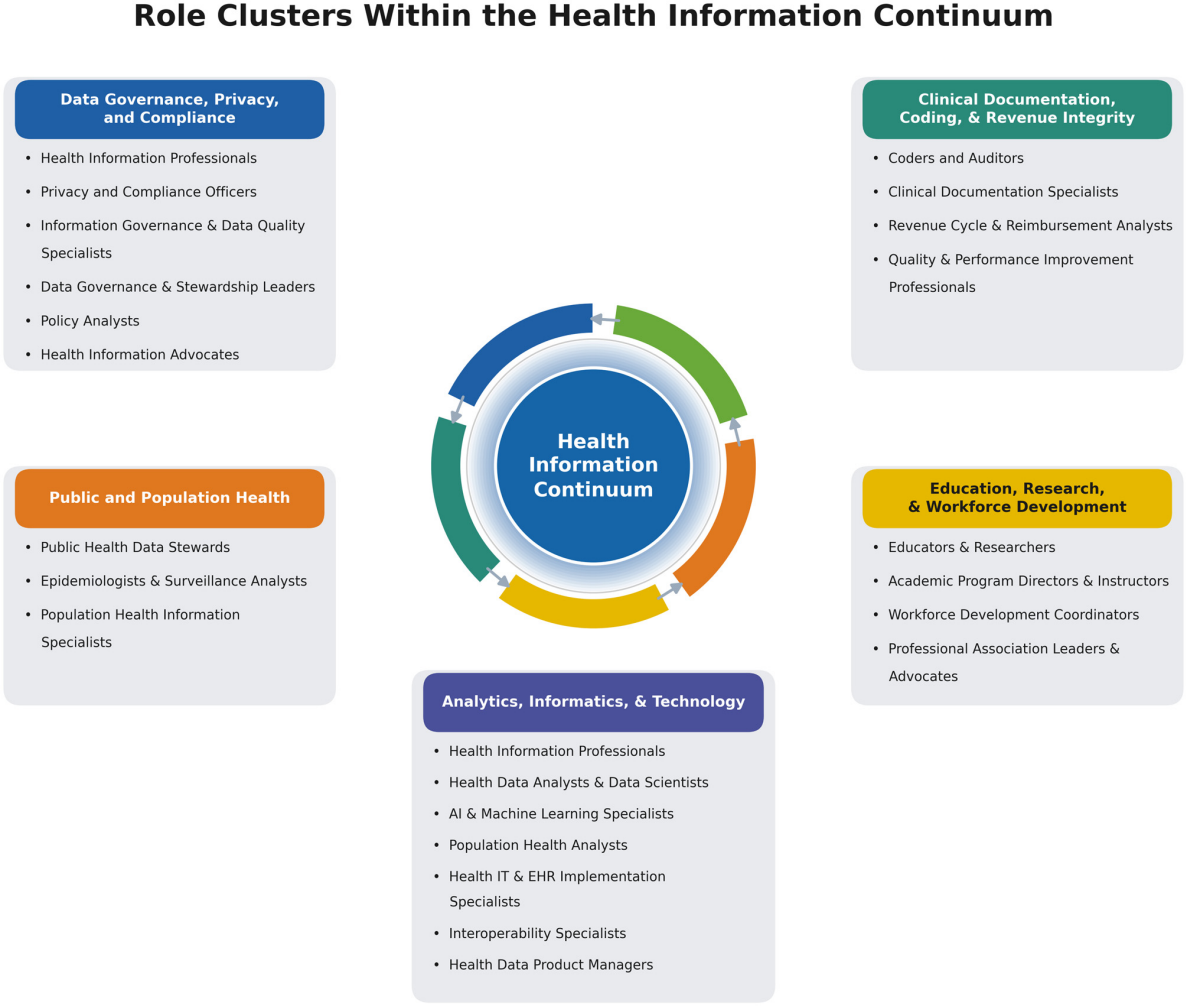


Role clusters within the Health Information Continuum illustrating functional groupings of HI work across governance, analytics, clinical documentation, public health, and education (Figure 3).

### Shared Principles Across the Continuum

Across all sectors, roles, and core elements, HI professionals rely on shared principles of information governance. Accountability, transparency, integrity, protection, compliance, availability, retention, and disposition apply regardless of setting. Whether managing clinical records, designing analytics platforms, supporting payer operations, or contributing to public health surveillance, these principles provide a consistent foundation for ethical and effective HI practice.^16^^,^^21^

### Why the Continuum Matters

Taken together, the Health Information Continuum provides a clearer and more accurate way to describe how HI work is organized, practiced, and valued across the health care ecosystem. By shifting the focus from setting-based labels to purpose, function, and data stewardship, the continuum offers a foundation for workforce development, education, and policy that aligns with the realities of modern HI practice.^2^^,^^3^^,^^10^

## Conclusion

The continued use of “traditional” and “nontraditional” labels no longer reflects how HI work is organized, practiced, or experienced. As demonstrated throughout this article, HI roles now span providers, payers, technology vendors, public health, education, research, and emerging digital health environments, with shared competencies and responsibilities that extend across settings. Persisting with setting-based language obscures this reality and reinforces outdated assumptions about where HI work belongs and how it should be valued.^2^^,^^3^

The findings presented here suggest that this language gap is not merely semantic. It influences professional identity, educational framing, workforce planning, and external understanding of the profession. When roles are described as deviations from an implied norm, the full scope of HI practice remains underrepresented, and the profession’s contributions are more easily misunderstood or minimized.^4^^,^^10^

The Health Information Continuum provides a clear and practical alternative. By organizing HI work around purpose, function, and the life cycle of health data rather than physical location, the continuum offers a more accurate and inclusive framework for describing the profession. It recognizes that all HI roles contribute to shared outcomes and that career mobility, interdisciplinary practice, and cross-sector collaboration are foundational features of modern HI work, not exceptions.^3^

Moving forward, educators, professional associations, employers, and workforce stakeholders are encouraged to move beyond the language of “traditional” and “nontraditional” roles and adopt continuum-based framing when describing HI practice. Although portions of the primary survey data were collected within a single state, these findings are interpreted alongside federal workforce data and national and international professional reports, supporting patterns that extend beyond a single geographic region. Looking ahead, expanding this work beyond Illinois at the state or national level offers an opportunity to better understand workforce distribution, emerging roles, and gaps across diverse settings. By replicating or adapting this approach through surveys, partnerships with component associations, and collaboration among educators, employers, and policymakers, the profession can build a broader, data-driven picture that supports clearer communication, more accurate workforce representation, and stronger alignment between education, practice, and policy. As HI continues to shape care delivery, population health, and system performance, the profession must also evolve the language it uses to describe itself. The Health Information Continuum offers a foundation for that shift.^2^^,^^10^

## Author Contributions

Both authors identified have contributed to this article.

## Disclosures

The authors have nothing to disclose.

## Funding

The authors received no funding for this research.

## Conflict of Interests

The authors declare that they have no known financial, professional, or personal conflicts of interest that could have influenced the research, analysis, or conclusions presented in this article.

CE Quiz

## Appendix A

**Table:** Data Sources and Measures

Data Source	Years	What it Contributes	Limitations
BLS SOC 29-2072, 29-9021	2024-2025	Workforce distribution	Excludes many HI roles
ILHIMA survey	2026	Practitioner insights	Regional sample
Educator survey	2026	Curriculum alignment	Small sample
AHIMA/NORC report	2023	National trends	Self-reported
HIMSS/ONC/HRSA	Various	Workforce ecosystem	Not HI-specific
